# Cost-effectiveness analysis of ambroxol for the treatment of Chinese patients with Gaucher disease

**DOI:** 10.3389/fmed.2025.1568709

**Published:** 2025-05-13

**Authors:** Yueyang Huang, Hongmei Yuan, Zhe Huang

**Affiliations:** School of Business Administration, Shenyang Pharmaceutical University, Shenyang, China

**Keywords:** Gaucher disease, imiglucerase, ambroxol, cost-effectiveness, Markov model, incremental cost-effectiveness ratio, sensitivity analysis

## Abstract

**Background:**

Gaucher disease (GD) is an autosomal recessive disorder with a wide range of clinical symptoms that cause abnormal function of parenchymal organs such as liver and spleen in patients. Enzyme replacement therapy, represented by imiglucerase, is a common approach for GD treatment. However, limited efficacy and high cost are important factors restricting its use. Ambroxol has gradually attracted attention due to its ease of administration, safety, and efficacy. However, there is no pharmacoeconomic evaluation of ambroxol for the treatment of GD in China. The cost-effectiveness profile of ambroxol combined with imiglucerase therapy for the treatment of GD, as opposed to imiglucerase monotherapy, needs to be investigated.

**Objective:**

This study aimed to analyze the cost-effectiveness of ambroxol for GD in China from the perspective of the Chinese healthcare system.

**Methods:**

We constructed an eight-state Markov model based on the disease characteristics of GD. The Markov cycle was 1 month. The time horizon was 6 years. The willingness-to-pay threshold was chosen to be 1–3 times the gross national product (GDP) per capita. The incremental cost-effectiveness ratio (ICER) was calculated from the base-case analysis, and one-way sensitivity analyses and probabilistic sensitivity analyses were performed.

**Results:**

The ICER value was ¥223,726.70, which was between 1 and 3 times GDP per capita. Sensitivity analysis showed that the cost of imiglucerase had a significant effect on ICER as well as demonstrating the stability of the results.

**Conclusion:**

Ambroxol combination therapy is a cost-effective regimen compared with imiglucerase monotherapy.

## 1 Introduction

Gaucher disease (GD) is a rare autosomal recessive disorder and the most common form of neurosphingolipid as well as lysosomal storage disease (1). It is primarily caused by a genetic abnormality that results in a deficiency of β-glucocerebrosidase (GBA), leading to a dramatic decrease in residual enzyme activity, which in turn causes an abnormal accumulation of glucocerebroside and the formation of Gaucher cells (2). Symptoms exhibited by patients with GD include hepatosplenomegaly, bone damage, disruption of lung homeostasis, decreased blood counts, growth retardation, and neurologic-related symptoms (3–6). It is estimated that the global incidence of GD is about 1/50,000–1/40,000, and the incidence of GD in China is about 1/200,000 (7). Currently, enzyme replacement therapy is a common approach for GD. One of the more classical drugs is imiglucerase (8). By injecting this drug, it can reduce the amount of hepatic parenchymal infiltration by Gaucher cells, which has a significant alleviating effect on liver fibrosis as well as portal hypertension (9). However, the inability of imiglucerase to cross the blood-brain barrier makes its efficacy very limited, and some complications such as Parkinson’s disease persist after treatment (10). Since the appearance on the market in China in 2009, imiglucerase has not been listed in the national medical insurance directory of China. Therefore, it has not been funded by the Chinese healthcare system. In addition, the price of imiglucerase is high, and the main source of the cost of imiglucerase replacement therapy is drug expense. It is obvious that high drug price will seriously affect the willingness of GD patients to treat with this therapy. Moreover, the cost of treatment increases with age, which can add to the burden on society and the patient’s family. Pharmacological chaperone therapy is a brand new way to treat GD. Pharmacological chaperones can selectively bind to misfolded GBA in the endoplasmic reticulum, promote the correct folding of proteins, and induce functional recovery (11). At the same time, small molecules of pharmacological chaperones can cross the blood-brain barrier and enhance the efficacy (11). Ambroxol, as an over-the-counter mucolytic agent, can act as a pharmacological chaperone for GBA. Currently, there have been studies demonstrating the promising efficacy of ambroxol as a pharmacological companion therapy, which, when used in conjunction with enzyme replacement therapy, has shown positive effects in patients with GD, specifically improvement in GD-associated hepatosplenomegaly, anemia, thrombocytopenia, chronic pain, and Parkinson’s disease (12, 13). As a therapy with high potential, an evaluation of its economic value is necessary. Currently, there is only one pharmacoeconomic study on ambroxol combined with enzyme replacement therapy regimen for GD worldwide (14), and more evidence is still needed to prove the economics of this therapy. Therefore, this study provides a cost-effectiveness analysis of ambroxol combined with imiglucerase enzyme replacement therapy from the perspective of the Chinese healthcare system to inform the decision-making of relevant healthcare organizations.

## 2 Materials and methods

### 2.1 Target population

Patients with type II and III GD (neuronopathic forms) who were born as well as those whose onset of disease began in infancy were selected as the study population.

### 2.2 Interventions and comparators

In this study, we chose imiglucerase monotherapy as the control regimen and the combination of imiglucerase and ambroxol as the intervention regimen. Imiglucerase was administered by injection at a dose of 2.5 U/kg three times per week. Ambroxol was administered orally at a dose of 25 mg/kg/day once daily (11). Based on the relatively early age of onset of GD and the lack of relevant body weight data, for this study, we assumed a mean patient weight of 20 kg.

### 2.3 Model structure

**FIGURE 1:**
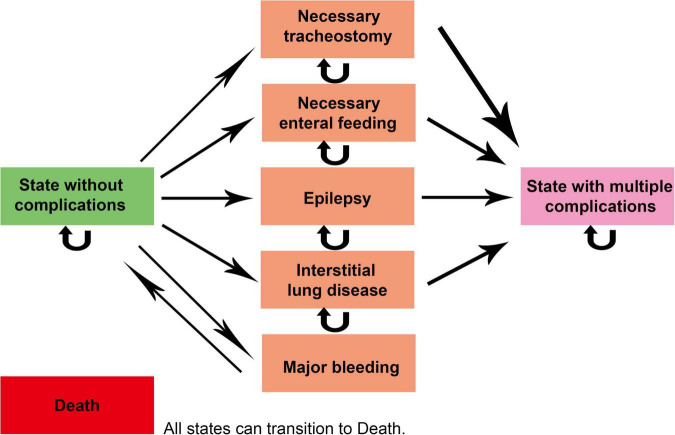
Markov model diagram. The status pointed by the straight arrow is the state after the transfer, and the reverse direction of the arrow is the initial state. The curved arrow indicates that the patient’s disease status has not changed during one cycle. The “Death” state (as an absorption state in the model) can be transferred from any of the other seven states.

Markov model was used in this study and the model diagram is shown in Figure 1. The model setup in this study was based on previous studies in Serbia as a reference (14). When modeling, the greater impairment of lung and other organ function and the negative effects of GD on blood function need to be taken into account (9), as well as the excellent efficacy of ambroxol in neurological improvement (12, 13). In addition, patients may have multiple complications as mentioned above, which need to be reflected in the model. Therefore, we set up eight states according to the disease characteristics of GD and the associated complications, including “State without complications,” “State with multiple complications,” “Necessary tracheostomy,” “Necessary enteral feeding,” “Epilepsy,” “Interstitial lung disease,” “Major bleeding,” and “Death” absorption status. The progression of the patient’s different states is represented by arrows in the figure. The state pointed by the arrow is the state after the transfer, and the state before the transfer is in the opposite direction. Due to the lack of initial state data from GD patients, we assumed that all patients entered the model in the state of “State without complications.” As can be seen from the arrow pointing between the various states in the figure, “State without complications” can be transformed into any other state in this model. Any other state in the figure (except “Death” state) can be transformed into the “Death” state.

Based on the natural course of GD and the survival time of the patients, we set the Markov cycle to 1 month (15), and the total model time horizon was 6 years (14, 16). In addition, we also performed a half-cycle correction (15).

### 2.4 Costs, utility values, and transfer probability data

The cost data, utility values, and data sources used in this study are presented in Table 1 as well as Table 2, respectively. Since the health system was chosen as the research perspective for this study, the costs included were direct healthcare costs, mainly in terms of drug costs. Due to the limited availability of data and the ruling proportion of drug costs in enzyme replacement therapy, other direct medical costs such as hospitalization and monitoring fees were not included in this research. The cost distribution was selected as normal distribution, and the transfer probability and utility value distribution were selected as beta distribution (17). Whereas, the transition probability was based on previously published studies (18–22), with reference to data from studies in other countries as well as extrapolations based on observational studies in the Chinese context, and the details are presented in Supplementary material. Among them, because the mortality data for each state were derived from the GD-related literatures (19, 20), the models in this study reflect the higher mortality rates of GD patients relative to the general population. In terms of adverse effects, since no serious adverse effects were found in patients with either drug, the additional costs incurred by them as well as the reduction in utility value were not considered in this study.

**TABLE 1:** Cost inputs.

Cost	Cost value	Distribution	Source
Imiglucerase cost (¥/400 U)	21,870	normal	www.menet.com
Ambroxol cost (¥/600 mg)	5.25	normal	www.yaozh.com

**TABLE 2:** Utility inputs.

Markov state	Utility value	Distribution	Source
State without complications	0.86	Beta	(14)
State with multiple complications	0.44	Beta	(14)
Necessary tracheostomy	0.68	Beta	(27)
Necessary enteral feeding	0.50	Beta	(28)
Epilepsy	0.55	Beta	(14)
Interstitial lung disease	0.55	Beta	(29)
Major bleeding	0.52	Beta	(30, 31)

### 2.5 Model outputs

The health output indicator for this study is quality-adjusted life years (QALYs). This is a standardized, generic health output metric that indicates the number of years a patient survives in perfect health. To reflect the difference in cost per unit of utility between the two treatment regimens, we used the incremental cost-effectiveness ratio (ICER), which is obtained by dividing the incremental cost by the incremental QALY totaled by the combined imiglucerase and ambroxol vs. the imiglucerase alone hypothetical cohorts of patients. We used 1–3 times the World Health Organization’s recommended gross domestic product (GDP) per capita as the willingness-to-pay (WTP) threshold. In 2023, China’s GDP per capita was ¥89,358. According to the China Pharmacoeconomics Evaluation Guidelines (2020 edition) (23), a discount rate of 5% was chosen for cost as well as QALYs in this study.

### 2.6 Sensitivity analyses

We used one-way sensitivity analysis and probabilistic sensitivity analysis (PSA), respectively. The former is to detect the influence of a single parameter on ICER, which is presented by up-regulating and down-regulating key parameters by 10%, so as to obtain the parameter with the strongest direct influence on ICER value (23, 24). The latter, on the other hand, simultaneously assesses the effect of uncertainty in all parameters on the results by performing 1,000 random samples of each parameter in the model which was given a theoretical probability distribution, and then presenting the simulation results on the cost-effectiveness plane (25) and plotting the cost-effectiveness acceptability curves (26). Due to the lack of corresponding data, we use 10% of the mean as the estimated range of variation in the standard error of the cost and utility values. Both sensitivity analyses were able to test the stability of the test results (17, 23, 24).

### 2.7 Analysis software

The Markov model in this study was performed in Treeage Pro2022 (2022; TreeAge Software; Williamstown, MA, United States), a professional decision tree and cost-effectiveness analysis software used in various industries such as healthcare.

### 2.8 Compliance with ethics guidelines

The data related to this study were obtained from clinical trials and previously published papers. This study did not contain any human or animal related experiments and therefore did not require approval from the Ethics Committee.

## 3 Results

### 3.1 Base-case results

The results of the base-case analysis are shown in Table 3. In terms of health outputs, when QALY was used as a measure, patients in the ambroxol group had a QALY value of 2.02, which was significantly higher than the QALY value of patients in the NU group (1.48). However, the ambroxol group also had to bear more costs. The ICER value derived from the deterministic analysis was ¥223,726.70/QALY, which was higher than 1x GDP per capita (¥89,358/QALY) and lower than 3x GDP per capita (¥268,074/QALY). This suggests that ambroxol combination therapy is an economical option to some extent relative to imiglucerase monotherapy.

**TABLE 3:** Base-case results.

Treatment	Cost (¥)	Incr cost (¥)	Eff (QALY)	Incr eff (QALY)	ICER (¥/QALY)
Imi	529,928.56		1.48		
Imi + Amb	650,629.56	120,701.00	2.02	0.54	223,726.70

Imi, imiglucerase; Amb, ambroxol.

### 3.2 One-way sensitivity analysis results

**FIGURE 2:**
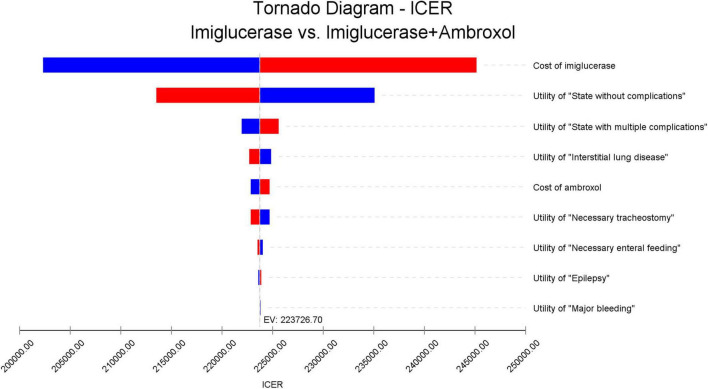
One-way sensitivity analysis results. The names of the parameters for which the analysis was performed are indicated on the right side of this figure and are listed in descending order of their effect on the ICER values. The red bar indicates the effect of increasing the corresponding parameter on the ICER value, and the blue bar indicates the effect of decreasing the parameter on the ICER value.

To test the stability of the results, this study conducted a one-way sensitivity analysis of the key parameters in the model. Finally, the impact of key parameter changes on ICER is presented in the form of a tornado diagram (Figure 2). The different directions of parameter changes in the figure are distinguished by red and blue colors, respectively. From the figure, it can be observed that the price of imiglucerase is the most influential factor on ICER, which reveals its important impact on the economics of the corresponding treatment regimen. Considering the high cost of enzyme replacement therapies, the large impact of imiglucerase cost on ICER values is to be expected. If imiglucerase is included in the national medical insurance directory in the future, its pricing will also be an important consideration in decisions related to healthcare budgets.

### 3.3 Probabilistic sensitivity analysis results

**FIGURE 3:**
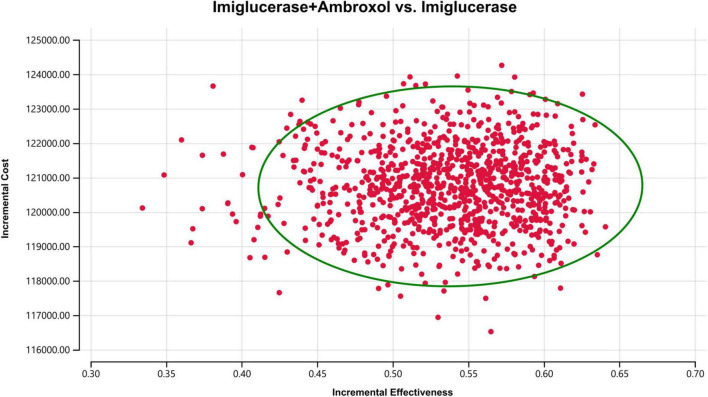
Cost-effectiveness plane for the imiglucerase + ambroxol vs. the imiglucerase group (WTP = ¥89,358/QALY). The X-axis represents incremental QALYs and the Y-axis represents incremental costs. The dashed line indicates the WTP threshold. In this figure, simulations in which ambroxol combination therapy is cost-effective are indicated by green dots (below the dashed line corresponding to the WTP threshold), and red dots (above the dashed line corresponding to the WTP threshold) if the opposite condition is reached.

**FIGURE 4:**
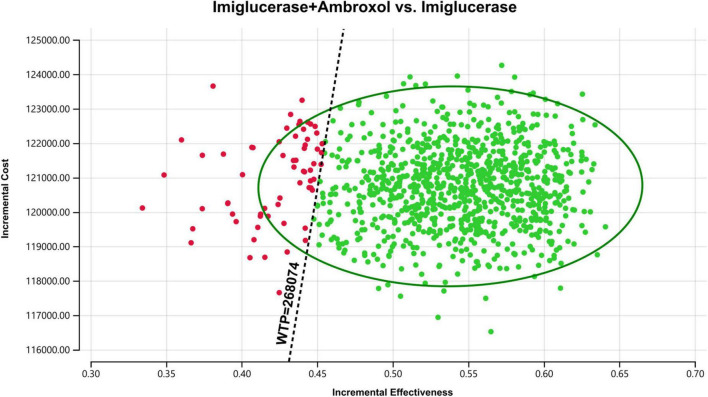
Cost-effectiveness plane for the imiglucerase + ambroxol vs. the imiglucerase group (WTP = ¥268,074/QALY). The X-axis represents incremental QALYs and the Y-axis represents incremental costs. The dashed line indicates the WTP threshold. In this figure, simulations in which ambroxol combination therapy is cost-effective are indicated by green dots (below the dashed line corresponding to the WTP threshold), and red dots (above the dashed line corresponding to the WTP threshold) if the opposite condition is reached.

**FIGURE 5:**
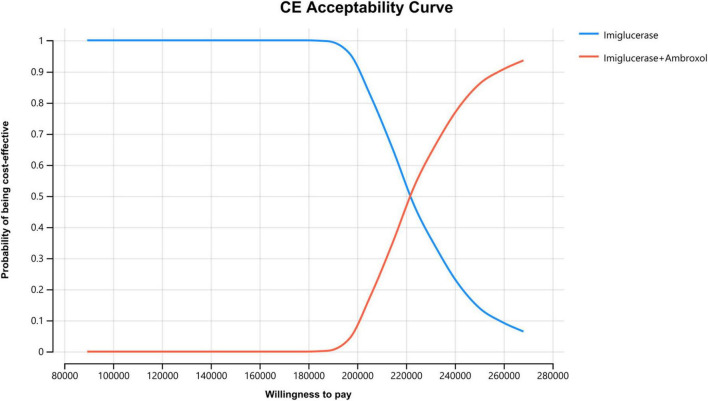
Cost-effectiveness acceptable curve. The X-axis represents the WTP value (from 1x GDP per capita to 3x GDP per capita) and the Y-axis represents the probability that the treatment program is cost-effective.

In order to assess the impact of parameter uncertainty on the results, we performed a PSA. The simulation results are presented on the cost-effectiveness quadrant plots as shown in Figures 3, 4. The x-axis represents the incremental QALY obtained and the y-axis represents the incremental cost. The green ellipse contains the 95% ICER estimate. We have chosen 1 and 3 times the GDP per capita as thresholds for the analysis, respectively. The results show that aminoglutethimide combination therapy resulted in more costs as well as additional effects in all simulations. The average ICER values were similar to the results of the deterministic analysis. In addition, we plotted the cost-effectiveness acceptable curve, and the results are shown in Figure 5. It shows the probability that two treatment options are cost-effective at a given threshold. We can find that as the willingness-to-pay threshold is adjusted upwards, the probability of having cost-effectiveness in the ambroxol group increases. At a threshold of 1x GDP per capita (¥89,358/QALY), the probability of the ambroxol group being cost-effective is 0. When the threshold rises to 3x GDP per capita (¥268,074/QALY), this value rises to 93.6%.

## 4 Discussion

As an autosomal recessive genetic disease, GD has a wide range of clinical symptoms and causes abnormal functioning of parenchymal organs such as liver and spleen in patients. Meanwhile, GD is also the most common neurosphingolipidosis and lysosomal storage disease, and is ranked 31st in the First Catalog of Rare Diseases published in China in 2018 (32). For GD, enzyme replacement therapy is currently approved for the treatment of GD, and it plays an important role in improving visceral, blood, and bone abnormalities (9, 10). However, the efficacy of this treatment in the nervous system is minimal (10, 11). In addition, in the case of limited efficacy, the cost of alternative therapies represented by imiglucerase is very high. In the background that imiglucerase has not been included in the list of medical insurance drugs in China, GD patients have to bear the vast majority of the cost of this treatment. The high expense is prohibitive for many GD patients. Ambroxol is a mucolytic sputum solubilizing agent cheaper than imiglucerase, and studies have been conducted to demonstrate the efficacy and safety of ambroxol in treating patients with GD (12, 33–35). In China, studies of long-term treatment with ambroxol have also been conducted, finding its safety in long-term treatment and suggesting that it is associated with clinical improvement in patients with GD (36). Therefore, our study is based on the Chinese background to evaluate the cost-effectiveness of ambroxol combined with imiglucerase therapy.

Our study found that ambroxol combined therapy was a cost-effective regimen, proved the stability of the results, and found that the cost of imiglucerase had an important effect on ICER value. We believe that the significant impact of the price of imiglucerase is in line with the current general concern of GD patients. What is clear is that ambroxol therapy trades an additional lower cost for a significant increase in patient QALYs. Considering the previously mentioned good ability of ambroxol therapy to cross the blood-brain barrier and the excellent therapeutic potential in terms of neurological improvement and so on, ambroxol combination therapy seems to be the more attractive option from a clinical and economic point of view. Our study did not include other patient scenarios provisionally, such as patients with longer treatment duration, patients with more severe complications, and patients with later GD diagnosis. It is likely that the benefits of ambroxol combination therapy will continue in these types of patients.

This study has several advantages. First, this study selected a popular therapy -ambroxol, as the study subject, which will provide a reference for subsequent studies. Second, this study is the first pharmacoeconomic evaluation of GD treatment in China, which is useful for the treatment of Chinese GD patients. Finally, the selection of Markov model is the key factor of the research. The Markov model used in this paper has a high degree of adaptation to GD, because each state can comprehensively reflect the disease progression of GD patients. Our study provides evidence support for the application of GD-related Markov model.

However, there are limitations to this study. First of all, as a typical rare disease, GD has few cases, high diagnostic difficulty and scattered distribution in China. Therefore, it is difficult to obtain utility values and the probability of disease metastasis related to GD patients in China, and no Chinese studies in this aspect have been carried out up to now. The utility value and transfer probability data used in this study are from studies in other countries, which may not fully reflect the actual situation of GD patients in China and may have a certain impact on the accuracy of the study. Taking the utility value as an example, this paper refers to the utility value of GD patients in Serbia. Same as a middle-income country, although the income level of residents in Serbia and China is similar, the local living conditions and medical policies will have an impact on the quality of life of the patients, and the accuracy of the utility value will be affected. Therefore, in addition to the relevant parameters for sensitivity analyses, the extent to which data such as utility values fit the Chinese context is another important aspect. As a key area of uncertainty, the impact of these data on the results should not be ignored. In addition, the effects of adverse reactions were not included in the modeling due to the lack of a reference standard, which may have created bias. Adverse reactions such as abdominal pain and anaphylactic reactions can actually occur with imiglucerase. Despite the small proportion and mild symptoms, they inevitably have a negative impact on patient utility values, treatment costs, and compliance. These biases will have an impact on the final results. Third, due to the lack of relevant data, the distribution of cost as well as utility in PSA used the mean ± 10%. Therefore, the uncertainty of the real situation may be different from that reflected in the PSA of this study. Furthermore, this study assumed that all patients entered the model as “State without complications,” which may not accurately represent the clinical diversity of GD patients. The diagnosis of patients with GD is still difficult, and it is inevitable that some patients will be misdiagnosed or diagnosed late. These patients are more likely to be treated for complications of GD. Furthermore, according to previous literature, the actual effective rate of ambroxol in the treatment of GD ranges from 29 to 100% (37). In this study, the effectiveness of ambroxol treatment was mainly reflected in the transition probability between Markov states. However, the effectiveness of ambroxol in GD patients in China remains unknown, and the existing data are likely to fail to reflect the real-world situation based on the Chinese background. Since there are relatively few patients with GD, whether ambroxol brings additional effects to patients with GD remains to be further studied. It also needs to be considered that due to the heterogeneity of GD, the degree of disease improvement in different GD patients using ambroxol varies. This study has limitations in data acquisition in this aspect (the lack of relevant data from China), and the treatment cost data and economic evaluation results may deviate from the real situation. Finally, this study still uses the more traditional 1–3 times GDP per capita as the willingness-to-pay threshold, which is likely no longer applicable to rare diseases represented by GD. Due to the small target population of rare diseases such as GD, the cost of their medication is difficult to be shared on a large scale, and thus the pricing of drugs is generally high. In addition, due to the narrow market space, even if the original drug has passed the patent period. There are also few generic versions, so it is difficult to reduce the price of drugs for rare diseases through competition. Such high prices result in many rare disease drugs not being able to meet the payment threshold of 1–3 times GDP per capita, making it difficult for them to be included in the medical insurance catalog. A large proportion of these medicines are of particular value in improving the quality of life of patients with rare diseases. In order to address the problem that the previous threshold (1–3 times GDP per capita) was too stringent for drugs for rare diseases, it is necessary and reasonable to raise the threshold appropriately. With regard to the economic evaluation of medicines for rare diseases, some scholars believe that the current payment thresholds are too biased in favor of the economic value of medicines, and that higher payment thresholds should be set for them, taking into account the severity of rare diseases, disease states and other factors (38). Some academics have also suggested additional funding impact assessments (39). We believe that the setting of rare disease-specific WTP thresholds is of great significance, allowing more expensive drugs to be included in the medical insurance catalog and improving their accessibility. Sensitivity analyses using rare disease-specific WTP thresholds can yield results that more accurately reflect the actual situation and provide valuable insights. We recognize that the above limitations may lead to very different conclusions. However, based on the limited data available, we believe that all data in this study were obtained from the best available sources, minimizing the impact of data bias on obtaining realistic conclusions.

It is worth noting that although ambroxol itself is less expensive, the price of imiglucerase used in conjunction with it remains high, which can lead to patients continuing to be influenced when considering combination therapies. Therefore, we recommend that manufacturers reduce the price of the drug or include it in the health insurance program to improve accessibility. In terms of drug use, high doses of ambroxol can maintain efficacy, but this may affect patient compliance (37), which is an essential aspect to be considered in the future. And patient compliance can have a direct impact on the cost and effectiveness of treatment regimens. Lower compliance, for instance, is likely to result in lower efficacy and higher long-term costs. In addition, there is still a lack of evaluative evidence on the economics of ambroxol therapy compared with other therapies for the treatment of patients with GD, which would undoubtedly drive the persuasive case for the economics of ambroxol therapy and drive it to clinical evaluation. Despite the advantages demonstrated in all aspects of ambroxol therapy, its prospective clinical trials remain lacking (22), in large part due to ambroxol’s own low cost. Therefore, the optimal dose of ambroxol for GD is still unknown. We call on relevant international organizations to collaborate to achieve an international consensus on GD treatment. As for pharmaceutical companies, Serbian scholars have pointed out that due to the low cost of ambroxol and its limited revenue potential, pharmaceutical companies are not as enthusiastic about clinical testing of ambroxol’s efficacy (14), which can greatly affect discoveries in GD treatment. We strongly agree with this viewpoint, and we believe that incentives from relevant healthcare systems for pharmaceutical companies to conduct clinical trials for rare diseases are necessary, including GD-related clinical trials. Finally, we call for the emergence of more clinical data based on GD patients in China. Relevant studies incorporating real-world data from the Chinese healthcare system are a possible area for future GD-related research, especially if there is sufficient local Chinese data on ambroxol therapy. The availability of more evidence of economic assessment based on the Chinese context will strengthen the conclusions we reached and will help to tailor future pharmacoeconomic models.

For the present GD-related therapies, the current Markov model used in this study is appropriate. It is likely that there will be emerging popular regimens regarding the treatment of GD in the future. We believe that dynamic modeling regarding pharmacoeconomic evaluation is likely to be applied in future GD-related research. As new clinical evidence continues to emerge, such dynamic modeling will provide a clearer and more specific perspective on the innovation of GD therapies. This study utilizes a healthcare system perspective, and some perspectives such as the social perspective are likely to provide additional insights. Considering the chronic nature of GD, the need for lifelong treatment, and the negative impact of certain disease states on patients’ work and life, an assessment from a societal perspective would yield a more specific and systematic assessment of relevant treatment options. For example, the inclusion of indirect costs in the social perspective takes into account the loss of productivity of the disease for patients and their families. In addition, as a member of a middle-income country, the conclusions of this study of the Chinese healthcare system may provide some reference for other middle-income countries, taking into account the relatively small differences in income levels and the clinical and economic advantages of ambroxol therapy itself. However, it should not be overlooked that, as a country with a population of more than 1.4 billion people, China has very limited healthcare resources per capita and imiglucerase has not yet been included in the medical insurance directory, which are factors specific to China. Therefore, other middle-income countries may refer to this study in the context of their own national conditions and drug policies. Moreover, as introduced earlier, not all GD patients have a good response to ambroxol. In actual clinical application and with reference to the results of this study, the treatment strategy needs to be adjusted in a timely manner in combination with the specific manifestations of the patients. We call for more studies on the therapeutic effectiveness of ambroxol based on the Chinese background in the future, which is crucial for subsequent economic evaluations.

## 5 Conclusion

In summary, from the perspective of the healthcare system, ambroxol is economical for the treatment of Chinese patients with GD. This study will help relevant organizations to make decisions, and the safety, convenience, and affordability of ambroxol are likely to make it dominant in the treatment of GD in the future.

## Data Availability

The datasets presented in this study can be found in online repositories. The names of the repository/repositories and accession number(s) can be found in the article/Supplementary material.
