# Supplementary material for: Cost-effectiveness analysis of ambroxol for the treatment of Chinese patients with Gaucher disease
Source: Front Med (Lausanne). 2025 May 13;12:1568709. doi: 10.3389/fmed.2025.1568709 (PMC12106307; doi:10.3389/fmed.2025.1568709)
Supplement: Supplementary file 1 [file Table_1.docx]

**Supplementary material**

1.Transfer probabilities

| Imi | A | B | C | D | E | F | G | H |
| --- | --- | --- | --- | --- | --- | --- | --- | --- |
| A | 0.8498 | / | 0.0224 | 0.0443 | 0.0224 | 0.0277 | 0.0324 | 0.001 |
| B | / | 0.9701 | / | / | / | / | / | 0.0299 |
| C | / | 0.1936 | 0.779 | / | / | / | / | 0.0274 |
| D | / | 0.2676 | / | 0.705 | / | / | / | 0.0274 |
| E | / | 0.2136 | / | / | 0.759 | / | / | 0.0274 |
| F | / | 0.2996 | / | / | / | 0.673 | / | 0.0274 |
| G | 0.9 | / | / | / | / | / | / | 0.1 |

Supplementary Table 1. Transfer probabilities of imiglucerase group.

| Amb+Imi | A | B | C | D | E | F | G | H |
| --- | --- | --- | --- | --- | --- | --- | --- | --- |
| A | 0.9196 | / | 0.0149 | 0.0165 | 0.0079 | 0.0185 | 0.0216 | 0.001 |
| B | / | 0.9741 | / | / | / | / | / | 0.0259 |
| C | / | 0.1146 | 0.864 | / | / | / | / | 0.0214 |
| D | / | 0.1456 | / | 0.833 | / | / | / | 0.0214 |
| E | / | 0.1846 | / | / | 0.794 | / | / | 0.0214 |
| F | / | 0.1266 | / | / | / | 0.852 | / | 0.0214 |
| G | 0.9 | / | / | / | / | / | / | 0.1 |

Supplementary Table 2. Transfer probabilities of Ambroxol+imiglucerase group.

Vertical is the transfer start state, horizontal is the transfer end state.

A: State without complications; B: State with multiple complications; C: Necessary tracheostomy; D: Necessary enteral feeding; E: Epilepsy; F: Interstitial lung disease; G: Major bleeding; H: Death
